# Dynamic iris biometry: a technique for enhanced identification

**DOI:** 10.1186/1756-0500-3-182

**Published:** 2010-07-01

**Authors:** Deborah M Rankin, Bryan W Scotney, Philip J Morrow, Douglas R McDowell, Barbara K Pierscionek

**Affiliations:** 1 School of Computing and Information Engineering, University of Ulster, Cromore Road, Coleraine, BT52 1SA, UK; 2 School of Biomedical Sciences, University of Ulster, Cromore Road, Coleraine, BT52 1SA, UK

## Abstract

**Background:**

The iris as a unique identifier is predicated on the assumption that the iris image does not alter. This does not consider the fact that the iris changes in response to certain external factors including medication, disease, surgery as well as longer term ageing changes. It is also part of a dynamic optical system that alters with light level and focussing distance. A means of distinguishing the features that do not alter over time from those that do is needed. This paper applies iris recognition algorithms to a newly acquired database of 186 iris images from four subjects. These images have greater magnification and detail than iris images in existing databases. Iris segmentation methods are tested on the database. A new technique that enhances segmentation is presented and compared to two existing methods. These are also applied to test the effects of pupil dilation in the identification process.

**Findings:**

Segmentation results from all the images showed that using the proposed algorithm accurately detected pupil boundaries for 96.2% respectively of the images, which was an increase of 88.7% over the most commonly used algorithm. For the images collected, the proposed technique also showed significant improvement in detection of the limbal boundary compared to the detection rates using existing methods. With regard to boundary displacement errors, only slight errors were found with the proposed technique compared to extreme errors made when existing techniques were applied. As the pupil becomes more dilated, the success of identification is increasingly more dependent on the decision criterion used.

**Conclusions:**

The enhanced segmentation technique described in this paper performs with greater accuracy than existing methods for the higher quality images collected in this study. Implementation of the proposed segmentation enhancement significantly improves pupil boundary detection and therefore overall iris segmentation. Pupil dilation is an important aspect of iris identification; with increasing dilation, there is a greater risk of identification failure. Choice of decision criterion for identification should be carefully reviewed. It needs to be recognised that differences in the quality of images in different databases may result in variations in the performance of iris recognition algorithms.

## Background

The function of the iris is to regulate the amount of light that enters the eye and reaches the retina. The regulation of pupil size is not only for controlling light levels, but is a response to optical, neurological and emotional factors mediated by the autonomic nervous system. Clinically, unless there is a neurological impairment or a neoplasm on the iris, this part of the eye receives less attention than other components. Iris conditions are relatively uncommon when compared to the range of anomalies and diseases that are found in the eye as principally ocular or as secondary manifestations of a systemic illness. However, beyond the clinical realm, the iris is increasingly becoming recognised as a tissue that can act as a reliable biometric for purposes of identification.

A biometric is any physical or behavioural characteristic that can be used to uniquely identify an individual. The suitability of a biometric is measured by the number of degrees-of-freedom or independent dimensions of variation. The iris contains approximately 266 degrees-of-freedom, the largest among facial features [1]. Uniqueness of the iris arises from its complex pattern that may contain many distinct features including nerve rings, fibre thinning, pigment spots and crypts. Each iris may also be classified according to texture (fine, fine/medium, medium/coarse, coarse) and colour (blue/grey, amber, light brown, brown, dark brown) [2].

The concept of the iris as a biometric means of identification was first proposed and patented by Flom and Safir [3]. Daugman [4,5] subsequently developed this system and his algorithm remains in use within many commercial iris recognition systems [6].

The importance of the iris as a unique identifier assumes that its appearance is stable throughout life and all biometric systems developed to date are based on this assumption. This does not take into account physiological changes to the iris, notably with age, as well as alterations to features that may occur in response to external factors such as medication, disease and/or surgery. The dynamics of the system also need to be considered as the iris expands and contracts with varying light levels and focussing distances.

Changes occur within varying time periods and depending on the extent of these changes, they may render iris recognition a less reliable method of identification than first proposed. What is required is a means of identifying the features in the iris which do alter over time from those that are immutable.

A reliable method for identifying iris features and distinguishing between those that alter and those that do not requires a data set with good quality images and development of algorithms to segment the iris, to extract pertinent features and to accurately match images of the same iris. Methods of segmentation and feature extraction vary in existing iris recognition algorithms [1,7]. This study is an extension of previous, preliminary work [8]. It considers the use of existing methods by applying them to an image data set with greater resolution of the iris than in previous databases and proposes an enhanced means of iris segmentation that would improve iris recognition and ultimately could help to better distinguish between mutable and immutable features. The effect of pupil dilation on iris recognition is also investigated.

## Methods

### Image capture

Images were captured using a Takagi clinical biomicroscope (slit lamp), model number SM-70 at 16 × magnification. Image size was 571 × 767 pixels with 96 × 96 dpi. The biomicroscope was attached to a desktop computer and Anterior Retinal Capture (ARC) specialist software was used to acquire, view and store images. Each subject focussed on a fixed target positioned on the slit lamp to maintain a steady primary gaze position each time images were captured and this was verified by stability in the position of Purkinje image I. Room lights were turned off to minimise spurious illumination and reflections. Slit lamp illumination was set to its lowest level so as to avoid discomfort to the subject and full constriction of their pupil. Slit beam angle was set at 45° and beam aperture was set at a maximum.

In total, 186 iris images from right eyes of four Caucasian adults aged between 23 and 64 years were captured: 19 images from Subject A were captured over 13 weeks, 98 from Subject B over 43 weeks, 40 from Subject C over 24 weeks and 29 images from Subject D over 16 weeks. Images were captured approximately 1-3 times per week for each subject. Tropicamide (0.5%), a standard clinical means of dilating the pupil that has a parasympatholytic action, was used on three separate occasions. This was to investigate whether increasing pupil size lowers iris image identification success rates as a change in pupil size alters the proportion of iris tissue that is visible. Tropicamide (0.5%) was instilled in the right eye of Subject B and 8 of Subject B's 98 images were captured when the pupil was dilated. Images taken for varying degrees of dilation were compared with images of the iris with a non-dilated pupil of the same subject. Ethical approval for the study was granted by the University of Ulster Biomedical Sciences Ethics Filter Committee.

### Image processing

#### *Segmentation*

**Figure 1:**
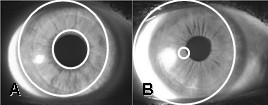
**Segmentation error rates**. The segmentation error rate is defined visually as (a) slight; and (b) extreme.

Iris segmentation required isolating the iris image by locating its inner (pupil) and outer (limbal) boundaries. The seminal techniques of Daugman [1] and Masek [7] were applied to the iris images collected in this study (described in detail below). Segmentation success is defined as the precision with which the pupil and limbal boundaries are located. The accuracy is determined by the difference in pixels between the area of the image segmented by the applied algorithm, and the area of the image segmented by expert visual assessment. In this study the accuracy was determined by visual inspection. There were only two categories of errors determined by visual inspection namely: slight error where the boundaries were slightly displaced by a few pixels (Figure 1 (a)) or extreme error where the boundaries were clearly displaced (Figure 1 (b)). It was found that these two categories were sufficient since all errors fell within these and so no further granularity was needed.

#### *Occlusion removal*

As part of Masek's algorithm, following inner and outer iris boundary detection, edge detection and thresholding algorithms were implemented to identify image regions containing occlusions from eyelids, eyelashes and specular reflections. These regions are discarded from further use in the latter stages of the algorithm.

#### *Normalisation*

**Figure 2:**
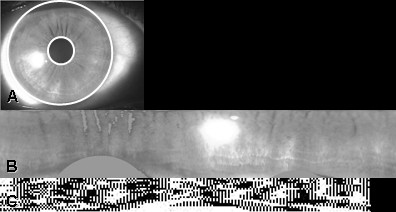
**Iris normalisation and encoding**. (a) Segmented image prior to normalisation and encoding; (b) Normalised iris showing iris remapped from Cartesian coordinates to a doubly dimensionless non-concentric polar coordinate system; (c) Encoded iris.

Normalisation is used to produce an iris image that has fixed dimensions and that is invariant to pupil dilation, scale, image size and camera-to-eye distance so that accurate comparisons can be carried out between iris images from the same person taken under varying conditions [1]. The homogenous rubber sheet model, which remaps the iris image from Cartesian coordinates to a doubly dimensionless non-concentric polar coordinate system, was used (Figure 2(a) and 2(b)).

#### *Feature extraction*

Pertinent features were extracted from the normalised iris pattern using log Gabor filters to decompose an iris image into complex-valued phase coefficients with amplitude information discarded^7^. These are quantised to obtain two binary values for each coefficient depending on the quadrant in which the value lies in the complex plane. An example of an encoded iris is shown Figure 2 (c).

#### *Matching*

The final stage requires comparison of different iris images to determine whether they belong to the same person. The test of statistical independence used involves calculating the Hamming Distance (HD) between two iris codes [1,7]. HD is a measure of dissimilarity between two irides and is based on a decision criterion derived from the bimodal distribution of inter-class (different subjects) and intra-class (same subject) variation of HDs calculated for many irides. The separation between these curves gives a range of values from which the decision criterion is selected. Values within this range were tested on iris images to determine how this affected identification.

Analysis was conducted on a subsample of 91 images, 2 of Subject A, 75 of Subject B, 9 of Subject C and 5 of Subject D with pupil dilation occurring in 7 of Subject B's images. These images were the ones accurately segmented by the proposed segmentation technique with occlusions removed successfully. The HD between all possible pairs of images was calculated giving 4186 pair-wise comparisons.

### Segmentation methods

#### *Daugman technique*

This method uses integrodifferential operators [1,4]. These are circular edge detectors used to locate the limbal and pupil boundaries of the iris. The image is smoothed with a Gaussian filter and the integral of the smoothed radial image derivative is computed along sequences of concentric circles centred at each pixel in the image. The maxima of these contour normal derivatives correspond to the pupil and limbal boundaries and are found using an exhaustive search across the image domain over all possible circles. Due to non-concentricity of the pupil and iris, separate searches were performed to detect the pupil and limbal boundaries, starting with the outer boundary. The primary search for the limbal boundary sets the smoothing function for a coarse scale of analysis due to the abrupt intensity transition between the iris and sclera. This first search is exhaustive across the image, while the second search looks only within the detected iris region to find the pupil boundary (the pupil will always be contained within the iris). The smoothing function is set to a finer scale of analysis in the pupil boundary search due to the fainter intensity transition between pupil and iris than between iris and sclera in Daugman's image dataset.

#### *Masek technique*

**Figure 3:**
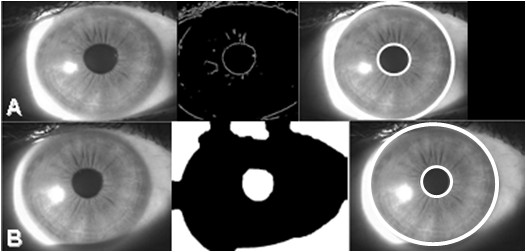
**Application of segmentation techniques**. Segmentation following: (a) Masek's technique showing image prior to segmentation; binary edge map obtained by the Canny operator; and resultant segmented image; (b) Daugman's technique with the proposed enhancement incorporated showing image prior to segmentation; thresholded image; and resultant segmented image.

Masek's algorithm [7] implements an edge detection operator [9] and a circular Hough transform [10] to segment the iris. This technique generates a gradient edge map using the Canny operator, which requires parameters to be supplied for use in the hysteresis thresholding stage of edge detection across different image data sets (Figure 3 (a)). The circular Hough transform is a standard computer vision algorithm for identifying and locating geometric objects from edge map information, and in this case the circular boundaries of the iris. The algorithm locates a circle of a given radius corresponding to the pupil or iris. Edge pixels (*x, y*) from the binary edge map are selected by first generating a 'circle of centres' for each edge pixel. The intersection point of the circles represents a maximum in terms of contrast variation and when completed for all edge pixels, the local maxima define the iris boundaries (pupil and limbal). The method searches for the limbal boundary first and then within the detected region for the pupil boundary (Figure 3 (a)).

#### *Proposed enhancement*

The proposed technique is an enhancement of Daugman's method. (This method was chosen because unlike Masek's algorithm [7], it does not require image dependent parameters). The Daugman method requires an exhaustive search of every pixel across the entire image domain. The proposed enhancement restricts the search to a limited region by finding an initial estimate of the pupil centre via thresholding the greyscale image [11]. Morphological operators are applied to the thresholded image and the pupil is differentiated from all other image objects as the main central object. It is detected by 'blob' analysis where a group of pixels organized into a structure, commonly called a blob, is analysed to obtain its characteristics: the centre of gravity and radius (shown in Figure 3 (b)). These values provide an estimate of pupil location within the image and are used to define a 10 × 10 search window within which precise pupil location is detected. Daugman's integrodifferential operators are applied in reverse order to initially detect pupil boundary followed by limbal boundary. When the pupil centre is found, a second search window is defined and Daugman's integrodifferential operator applied to detect the limbal boundary (Figure 3 (b)).

## Results

### Segmentation

When the results for segmentation success are compared, they show that the technique of Masek locates the pupil boundary in 67.2% of images in this study; the iris boundary is detected in only 14% of images (Table 1). Of the 186 images used in experimentation, both pupil and iris boundary were successfully detected in just 3.2% of the images using Daugman's technique. The inner boundary was detected in 7.5% and the outer boundary was detected in 12.4% of the images.

**Table 1:** Success rates for pupil and iris boundary detection

	Pupil		Iris		Pupil and iris	
**Method**	**No**.	**%**	**No**.	**%**	**No**.	**%**

**Masek**	125	67.2	27	14.5	26	14

**Daugman**	14	7.5	23	12.4	6	3.2

**Proposed**	179	96.2	110	59.1	109	58.6

Using the proposed method of enhancement, accuracy in pupil detection using Daugman's method increased to 96.2%, an increase of 88.7% over Daugman's original method. This method also provided a 29% improvement on Masek's pupil detection technique from 67.2% to 96.2%.

**Figure 4:**
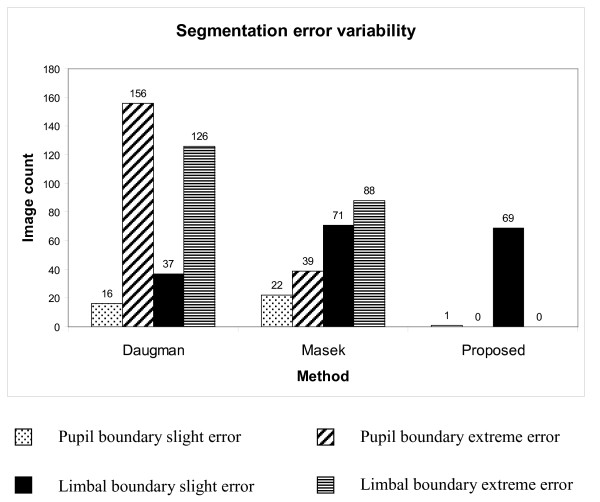
**Segmentation error rates**. Variability of segmentation error rates across the methods of Daugman, Masek and the proposed enhancement to the method of Daugman.

Results show that the proposed enhanced segmentation algorithm produces only slight errors in all cases of segmentation failure; there are no extreme errors (Figure 4). In comparison, Masek's technique resulted in a variety of slight and extreme in accuracies across images. For the pupil boundary, extreme inaccuracies were found only for images from blue and green eyes. Both slight and extreme segmentation inaccuracies, irrespective of eye colour, were found for the limbal boundary. Daugman's technique showed predominantly extreme segmentation inaccuracies throughout the data sets for both pupil and limbal boundary detection.

### Pupil dilation-matching criteria

**Figure 5:**
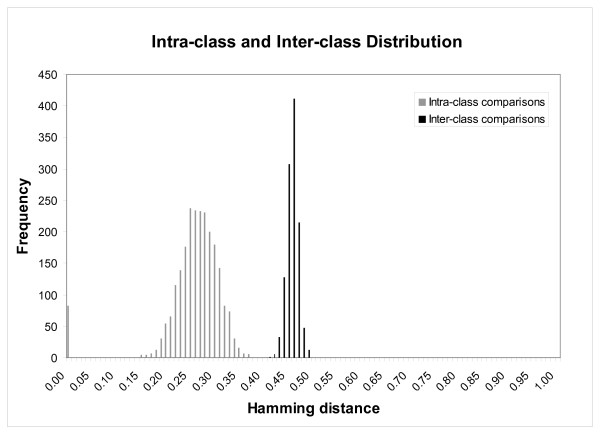
**Hamming distance distributions**. Intra-class (same subjects) and inter-class (different subjects) comparison distribution showing separation between maximum intra-class HD = 0.37 and minimum inter-class HD = 0.41.

A combination of the proposed segmentation technique developed in this study along with the occlusion removal, normalisation, feature extraction and encoding, and matching sections of Masek's iris recognition algorithm was used [7], to determine iris the effect of pupil dilation on iris identification. In order to determine the criterion against which the matching decision was to be made (i.e. whether a pair of images were considered to be from the same iris), the HD distribution of inter-class and intra-class comparisons was plotted. This is shown in Figure 5. The decision criterion is chosen based on the separation between intra-class and inter-class distributions. Intra-class images have a low HD as these images are of the same person, whereas inter-class (different subjects) comparisons will produce high HD values. A point between the two distributions is chosen to characterise the difference between the inter- and intra-class distributions, and to set the decision criterion about whether two images are from the same individual. Figure 5 shows that the maximum intra-class comparison HD is 0.37 and the minimum inter-class comparison HD is 0.41. HD criteria between these two values were tested to determine identification success for the dilated images.

Images featuring pupil dilation from Subject B were compared with images from the same person without dilation. This consisted of 476 comparisons (68 non-dilated images compared with each of the 7 dilated images). Of the 7 dilated images, 3 were slightly dilated (4.6 mm), 3 were moderately dilated (6.3 mm) and 1 was highly dilated (7.4 mm).

Results in Table 2 show that pupil dilation at 4.6 mm typically does not cause identification failure except in one case where the decision criterion was set to a value of 0.37. As pupil dilation increased, identification success decreased. For a dilation of 7.4 mm, high rates of identification failure are seen (Table 2); with least failures when the decision criterion is 0.41 where just over half the images are identified correctly for high dilation.

**Table 2:** Dilated pupil identification success results

	Identification success (%)					
**Decision criterion HD**	**0.37**	**0.38**	**0.39**	**0.40**	**0.41**	**Total images**

**4.6 mm dilation**	99.5	100	100	100	100	204

**6.3 mm dilation**	84.3	91.2	96.6	99.5	100	204

**7.4 mm dilation**	4.4	14.7	20.6	36.8	57.4	68

## Discussion

### *Segmentation*

For an iris recognition system to identify iris images accurately, precision is required at every stage of processing. This study has considered segmentation and matching as applied to dilated and undilated pupils. Specific segmentation methods that function successfully on certain data sets have been proposed but as yet there is no generic technique that works well on all datasets which contain images taken under varying conditions. Images collected in previous studies, CASIA [12] and Bath [13] are of lower magnification and resolution than those used in this study, and so contain less feature detail. They also contain a significant portion of surrounding facial detail and have fewer iris pixels. The images presented in this study have been collected at higher magnification using specialised clinical imaging equipment.

Results from this study show that Daugman's method has the worst performance in detecting both pupil and iris boundaries for the image dataset collected in this study.

Daugman [1,4] introduced integrodifferential operators that behave as circular edge detectors to detect the limbal and pupil boundaries of the iris by computing the maxima in the contour integral of a smoothed radial image derivative along concentric circles. A major factor in the poor performance of Daugman's technique is that if the limbal boundary is not accurately located initially there are difficulties with pupil detection. Masek's technique was found to be relatively more effective in pupil detection but could be improved for limbal boundary detection. The proposed method of enhancement significantly improved detection of both pupil and limbal boundaries. It should be noted that the images in this database of a much higher quality than those used in previous studies. The performance of the tested algorithms may depend on the quality of the images. Hence the lower performance found for the existing algorithms of Daugman and Masek, compared to the enhancement proposed, may reflect the fact that these algorithms were developed with reference to databases of lower quality images.

Enhancements to Daugman's method have also been proposed by other authors. Tisse [14] implemented a combination of integrodifferential operators and the circular Hough transform to obtain an approximation of pupil centre and to provide an improved starting point for the integrodifferential operator. This technique improved segmentation accuracy by 14% on a database of 50 eye images from 5 subjects. The database used by Tisse [14] was of lower resolution and magnification than the images used in this study. Zuo et al [15] proposed a segmentation technique in which intensity and location characteristics of the pupil and iris were enhanced before segmentation using Daugman's technique, and this was compared to Masek segmentation. Using images from the CASIA database, this enhancement to Daugman's technique reported an increase of 12.84% on segmentation success when compared to Masek's technique.

An alternative segmentation method, first proposed by Wildes [16], utilises an edge detection operator and the Hough transform to detect the circular boundaries of the iris. Masek [7] implemented a similar technique but with Canny edge detection.

Techniques that use integrodifferential operators remain the most robust of segmentation methods as they do not require definition of parameters across different image data sets, but enhancements are still required. Hough transforms have provided a feasible alternative but require image dependent parameters to be defined that may alter when capture conditions change. A technique that functions generically across all imaging conditions has not yet been developed.

#### *Pupil dilation-matching*

Matching to decide if a pair of iris images are from the same individual is conducted by obtaining the HD between two irides and determining whether they match based on a chosen threshold. This threshold value is derived from the bimodal distribution of inter-class and intra-class variations. This value selection is to some extent subjective and in practice the choice depends on the desired security level. A high security system will have a low decision criterion to ensure little chance of a false match. This will, however, increase the possibility of rejecting a match between iris image pairs from the same individual. Conversely, for a low security system, the decision criterion could be increased to reduce the number of false rejections but will in turn increase the possibility of incorrectly matching iris images from different individuals. A decision criterion of HD = 0.4 has been used previously [1,7]. In this study, a range of HD values between 0.37 and 0.41 were tested for a series of pupil dilations. The success in matching can be improved by selecting a higher HD value, but success rate decreases rapidly regardless of HD value for a pupil size above 7 mm. Further work is required to develop a system that can recognise the changing behaviour of iris structure caused by pupil dilation.

Variability in iris pattern and structure may differ according to varying factors, e.g. light and medication may deform the iris differently [17]. The effect of other causes such as surgical procedures and medical conditions, as well as age, may result in changes to the iris over time. A system that can accurately match a pair of iris images from the same individual irrespective of changes in image features will require enhancements at all stages of processing together with a means of knowing which features are immutable.

## Conclusions

The robustness of iris segmentation techniques have been examined on a new database of high quality images. Existing methods produced inadequate results when applied to the images collected in this study. A proposed enhancement of one of the existing techniques improves segmentation accuracy significantly and will advance the development of iris recognition systems and assist in identification of feature stability. Matching reliability is significantly lower when a pupil is dilated. A more thorough investigation of iris dynamics across a larger population is required.

## Competing interests

The authors declare that they have no competing interests.

## Authors' contributions

DMR undertook collection of the data, conducted the analysis, and contributed to writing the paper; BWS and PJM were involved in initiation of the project, conducting analysis and contributing to writing the paper, DRM helped with data collection, BKP was involved in initiation of the project, clinical data analysis and writing of the paper. All authors read and approved the final manuscript.
